# CRISPR-Cas12a based internal negative control for nonspecific products of exponential rolling circle amplification

**DOI:** 10.1093/nar/gkaa017

**Published:** 2020-01-20

**Authors:** Bo Tian, Gabriel Antonio S Minero, Jeppe Fock, Martin Dufva, Mikkel Fougt Hansen

**Affiliations:** 1 Department of Health Technology, Technical University of Denmark, DTU Health Tech, Building 345C, DK-2800 Kongens Lyngby, Denmark; 2 Blusense Diagnostics ApS, Fruebjergvej 3, DK-2100 Copenhagen, Denmark

## Abstract

False-positive results cause a major problem in nucleic acid amplification, and require external blank/negative controls for every test. However, external controls usually have a simpler and lower background compared to the test sample, resulting in underestimation of false-positive risks. Internal negative controls, performed simultaneously with amplification to monitor the background level in real-time, are therefore appealing in both research and clinic. Herein, we describe a nonspecific product-activated single-stranded DNA-cutting approach based on CRISPR (clustered regularly interspaced short palindromic repeats) Cas12a (Cpf1) nuclease. The proposed approach, termed Cas12a-based internal referential indicator (CIRI), can indicate the onset of nonspecific amplification in an exponential rolling circle amplification strategy here combined with an optomagnetic readout. The capability of CIRI as an internal negative control can potentially be extended to other amplification strategies and sensors, improving the performance of nucleic acid amplification-based methodologies.

## INTRODUCTION

Although nucleic acid amplification plays a vital and basic role in both research and clinic, its accuracy depends highly on the awareness of risk factors and the control of false-positive and false-negative results (1–3). The most sensitive nucleic acid amplification strategies are those employing exponential amplification formats in which amplicons (amplification products) are recycled as primers or templates (4). However, due to the exponential format, nonspecific background products (NBPs) that lead to false-positive results are inevitable after long reaction times and can be caused by, e.g., contaminants, off-template polymerase products, and secondary structures of primers or templates (4,5). Therefore, the reaction time of exponential amplification has to be evaluated and controlled in practice to avoid the influence of NBPs (5–7). Consequently, the sensitivity of exponential amplification based diagnostics is always determined by the resolution between true- and false-positive signals generated by diluted standard samples and blank/negative controls, respectively. Many efforts have been made to postpone the NBP generation. For example, Reid *et al.* applied single-stranded DNA-binding protein to reduce nonspecific template interactions in a nicking-based exponential amplification reaction and lowered the background by three orders of magnitude (8); Ding *et al.* utilized two pairing-competition primers in loop-mediated isothermal amplification to suppress the NBP generation during 2-h incubation (9).

In amplification strategies that recycle amplicons as templates (e.g. recombinase polymerase amplification), NBPs have different sequences compared to the desired specific products, suggesting that amplification results can be confirmed by electrophoresis and gene sequencing. However, in strategies that recycle amplicons as primers (e.g. primer generation-rolling circle amplification [PG-RCA]), NBPs are nearly identical to specific products, which means that the quality of the results depends highly on the performance of external negative controls. External negative controls, usually culture medium or samples from a control group of healthy individuals, are performed in parallel in nucleic acid diagnostics to indicate the generation of NBPs and hence the safe time range for a true-positive assessment (3). The application of external negative controls is based on the assumption that the control and test samples contain the same components (except for the specific target) and that differences, if any, have no influence on the final judgement. However, external negative controls usually have a simpler and lower background compared to the test sample. This is particularly true for the common practice of using water or buffer solutions as blank controls, and results in underestimation of false-positive risks. Moreover, external negative controls are required in each relevant clinical diagnostic case for reliable results, which increases not only the cost of detection but also the complexity of designing automated nucleic acid analysis. Due to these inherent shortcomings of external controls, reliable internal negative controls are urgently needed in research and in clinic.

Herein we report a CRISPR-Cas12a-based NBP-activated molecular strategy that digests all single-stranded DNAs (ssDNA) including the amplicons, and therefore suppresses the output signal upon NBP generation. This approach, named Cas12a-based internal referential indicator (CIRI), is performed together with PG-RCA (10) to indicate the level of nonspecific amplification for the tested samples. PG-RCA initiates by the hybridization between target sequences and circular templates (detection loops, DL). Target sequences are extended as primers on the DL by phi29 polymerase, generating amplicons that are subsequently nicked and strand-displaced. Since released single-stranded amplicons can hybridize with DL and serve as primers, PG-RCA achieves an exponential amplicon production with time.

CIRI is applicable when nonspecific amplification products contain a Cas12a activation sequence. In addition to PG-RCA, CIRI can be implemented in (i) exponential amplification schemes that recycle amplicons as primers, e.g., bidirectional strand-displacement amplification (11) and exponential amplification reaction (EXPAR) (12); (ii) cascade amplification schemes containing a step utilizing amplicons of the previous step as primers or triggers, e.g., hairpin-mediated quadratic enzymatic amplification (13); and (iii) cascade amplification schemes containing an RCA step, e.g., circle-to-circle amplification (14).

## MATERIALS AND METHODS

### Chemicals and DNA sequences

Phi29 polymerase, phi29 buffer, dNTP mix, bovine serum albumin (BSA), GeneRuler low range DNA ladder, SYBR Gold nucleic acid gel stain, Tris-HCl buffer (1 M, pH 8.0) and Tris-acetate-EDTA buffer (TAE, 50 ×) were purchased from Thermo Fisher Scientific (Waltham, MA, USA). CircLigase II ssDNA ligase, together with other ligation reagents (buffer, MnCl_2_, betaine), was purchased from Biosearch Technologies (Novato, CA, USA). Nb.BtsI nickase, thermolabile exonuclease I, exonuclease III and loading buffer were purchased from New England BioLabs (Ipswich, MA, USA). Recombinant *Acidaminococcus sp. BV3L6* Cas12a nuclease was purchased from Integrated DNA Technologies (Coralville, IA, USA). Fetal bovine serum (FBS), salmon sperm DNA and agarose were purchased from Sigma-Aldrich (St. Louis, MO, USA). Streptavidin-coated cross-linked starch iron oxide composite particles (100 nm size MNP) were purchased from Micromod Partikeltechnologie GmbH (Rostock, Germany). DNA and RNA sequences were synthesized by Integrated DNA Technologies and diluted in 50 mM Tris-HCl (pH 8.0). Sequences of targets (target dengue sequence and target B for amplifying detection loop and reference loop, respectively), linear templates (linear detection template and linear reference template), crRNA and detection probes (DP-DL-I, DP-DL-II, DP-RL-I and DP-RL-II) are listed in Supplementary Table S1.

### Preparation of circular templates

Circular templates, DL (detection loop) and RL (reference loop) were prepared by self-ligation of linear detection template and linear reference template, respectively. The ligation mixture consisted of linear templates (0.5 μM), CircLigase II reaction buffer (1×), MnCl_2_ (2.5 mM), betaine (1 M) and CircLigase II ssDNA ligase (5 U/μl). The ligation mixture was incubated at 60°C on a ThermoShaker incubator (Grant-bio, Cambridge, UK) for 3 h to form circular templates, followed by heat inactivation at 80°C for 15 min. To remove remnant linear templates, exonuclease I and exonuclease III were added to the solution to final concentrations of 0.5 and 5 U/μl, respectively. The digestion was conducted at 37°C for 1 h, followed by heat inactivation at 80°C for 15 min.

### Functionalization of magnetic nanoparticles

Tris-HCl (50 mM, pH 8.0) was used for MNP washing (with a magnetic stand) and resuspension. Streptavidin-coated 100 nm MNPs were washed twice and resuspended to 1 μg/μl before conjugation. Biotinylated detection probes were added into the MNP suspension to a concentration of 0.5 μM. The suspension was incubated at 37°C for 30 min, washed three times to remove unbound detection probes, and resuspended to an MNP concentration of 1 μg/μl. For the detection of DL amplicons, DP-DL-I modified MNPs and DP-DL-II modified MNPs were prepared separately, mixed in a volumetric ratio of 1:1, and stored at 4°C prior to use. For the detection of RL amplicons, DP-RL-I modified MNPs and DP-RL-II modified MNPs were prepared, mixed and stored in the same way.

### Primer generation-rolling circle amplification

For CIRI-controlled PG-RCA, the RCA reaction mixture consisted of phi29 buffer (2×), BSA (0.4 μg/μl), dNTPs (0.45 mM), phi29 polymerase (0.67 U/μl), Nb.BtsI nickase (0.2 U/μl), Cas12a (60 nM), crRNA (90 nM), circular templates (1 nM each of DL and RL) and detection probe-modified MNPs (0.1 μg/μl). Upon detection, the RCA reaction mixture was mixed with the test sample in a volumetric ratio of 1:1, followed by isothermal incubation at 37°C on chips (for real-time optomagnetic measurement). For PG-RCA reactions performed without CIRI, Cas12a was excluded from the RCA reaction mixture. For preparing the blank control, 50 mM Tris-HCl (pH 8.0) free of target sequence was mixed with the RCA reaction mixture in a volumetric ratio of 1:1. In the utility test using cell DNA extractions, 50 mM Tris-HCl (pH 8.0) containing 0.1 μg/ml sheared salmon sperm DNA was used to prepare the blank control.

### Preassembly of MNP clusters

The RCA reaction mixture (without CIRI, MNP concentration of 0.5 μg/μl) was mixed with a sample containing 2 pM of target DNA in a volumetric ratio of 1:1, followed by isothermal incubation at 37°C for 1 h. After PG-RCA reaction, MNP clusters formed in the suspension were collected by a magnetic stand, washed twice and resuspended to an MNP concentration of 0.1 μg/μl by Tris-HCl (50 mM, pH 8.0).

### Real-time optomagnetic measurement with magnetic incubation

Optomagnetic detection chips (a minimum detection volume of 90 μl) containing RCA suspensions were sealed and thereafter mounted in the optomagnetic setup. Ten magnetic incubation cycles were automatically performed between two optomagnetic measurements, with each cycle consisting of 2 s of 2.6 mT field followed by 2 s of 0 mT. For each optomagnetic measurement, an optomagnetic spectrum consisting of 41 logarithmically equidistant frequencies between 1 and 2800 Hz was recorded in *∼*50 s.

### Agarose gel electrophoresis

In the gel electrophoretic study, concentrations of circular templates, targets and Cas12a-crRNA used in the PG-RCA reaction were 0.5 nM, 1 pM and 30 nM, respectively. PG-RCA reactions were conducted at 37°C for 30 min. Products of PG-RCA were mixed with loading buffer as well as SYBR Gold gel stain, and analyzed by 2.5% (w/v) agarose gel electrophoresis in 1 × TAE buffer at a constant voltage of 100 V for 30 min at room temperature. Gel images were obtained by using a UVP BioSpectrum Imaging System (Analytik Jena, Jena, Germany).

## RESULTS AND DISCUSSION

**Figure 1.**
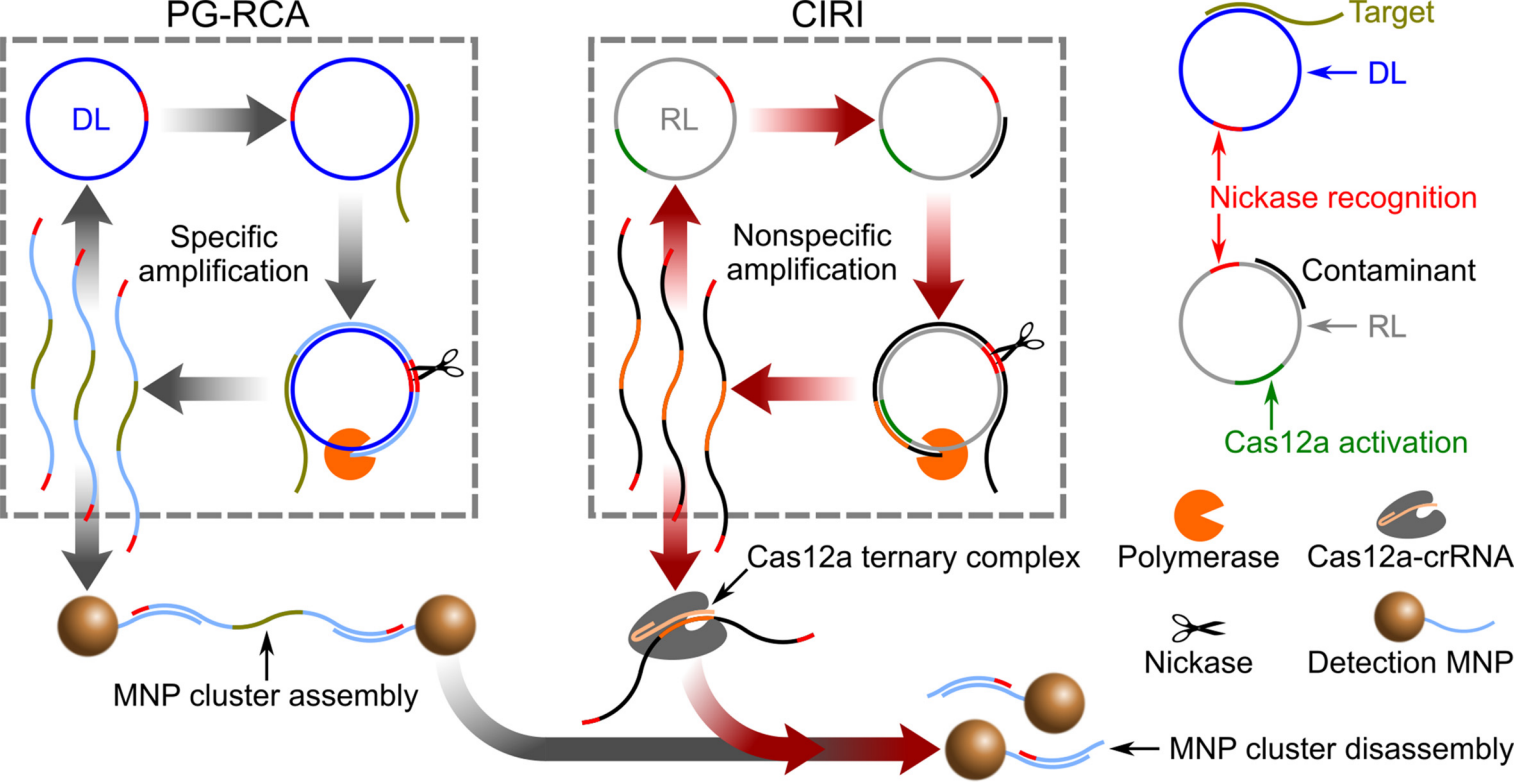
Schematic illustration of CIRI-controlled PG-RCA. Gray arrows indicate PG-RCA reactions that lead to the assembly of MNP clusters. Red arrows indicate activated CIRI that results in disassembly of MNP clusters.

In this work, amplicons of PG-RCA hybridize with dual detection probe-modified magnetic nanoparticles (MNPs) such that MNP clusters form in the presence of DL amplicons (Figure 1, gray arrows). The clustering of MNPs is monitored in real-time by an optomagnetic sensor. A detailed underlying theory of the optomagnetic sensor is provided in Supplementary Section S1 as well as in our previous work (15–18).

As an internal reference, CIRI employs another circular template for RCA (reference loop, RL) and an RNA-guided DNase, CRISPR-Cas12a. Upon activation, Cas12a indiscriminately cleaves ssDNA, which can be utilized for nonspecific degradation of reporter DNA and thus for biosensing (19–23). In our approach, activated Cas12a cleaves the DL amplicon links between MNPs, leading to MNP cluster disassembly. RL has no sequence overlap with but the same GC content and length as DL. In addition, RL includes a DNA sequence identical to the 20-nt-long recognition segment of crRNA (CRISPR guide molecule), which means that amplicons of RL can be recognized by crRNA and thereby activate Cas12a. Once RL is activated, NBPs produced by RL are recognized by crRNA to form catalytic ternary complexes with Cas12a, which leads to an optomagnetic signal reduction due to the disassembly of MNP clusters (Figure 1, red arrows). Therefore, the Cas12a-based signal reduction can be used as an internal indicator for false-positive reactions in a real-time nucleic acid analysis. In this study, we used CIRI-controlled PG-RCA for the detection of a synthetic DNA target representing a highly conserved genome sequence from all four different serotypes of dengue virus (24).

**Figure 2.**
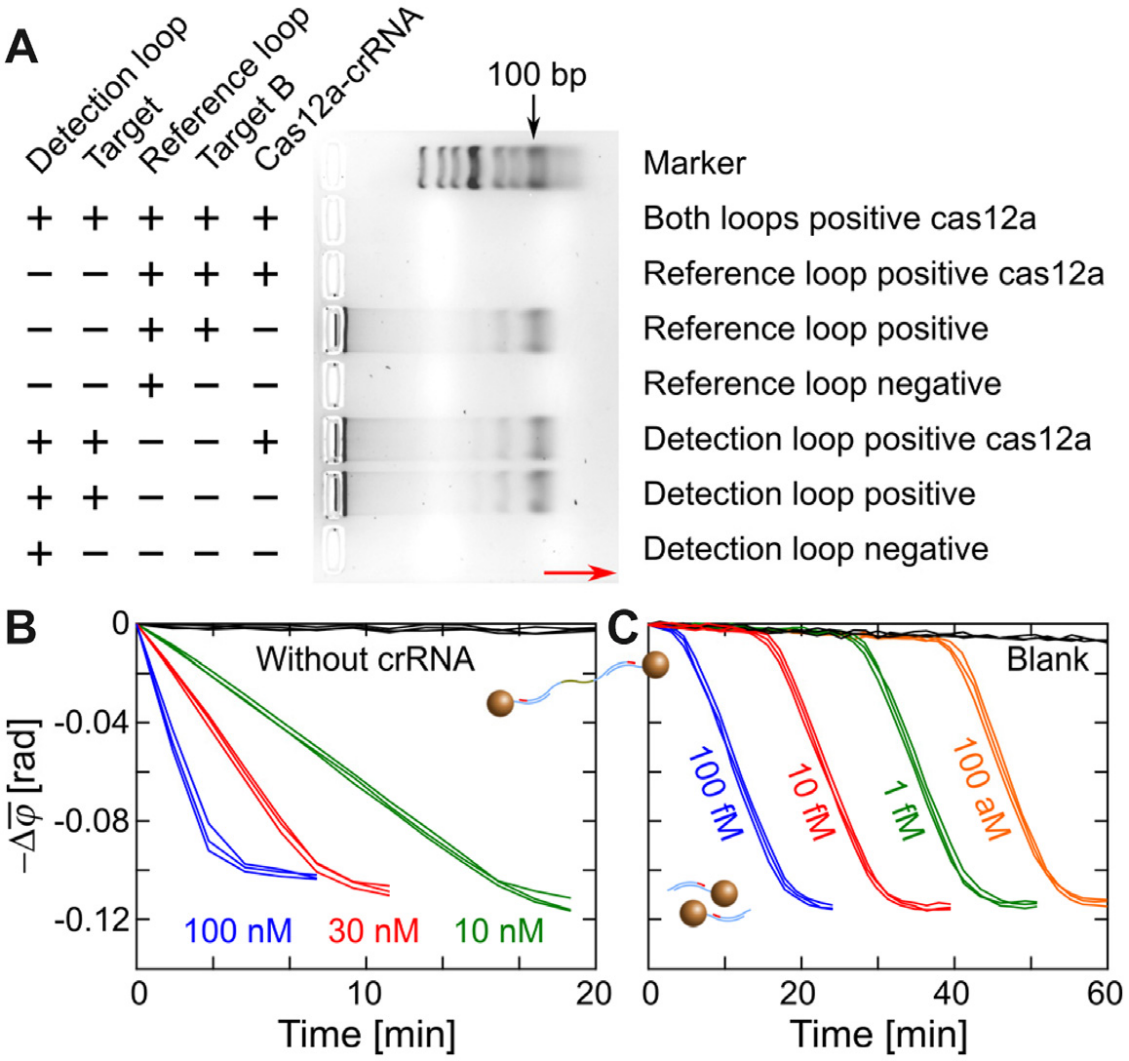
Examination of Cas12a for its capability of nonspecific trans-ssDNA cleavage and compatibility with PG-RCA. (**A**) Agarose gel electrophoresis analysis for the PG-RCA and CIRI. The red arrow indicates the direction of electrophoresis. (**B**) Real-time changes of $\bar{\varphi }$ (1–10 Hz) for disassembly of MNP clusters by Cas12a ternary complexes. Working concentrations of Cas12a are indicated in the plot. (**C**) Real-time changes of $\bar{\varphi }$ (1–10 Hz) for disassembly of MNP clusters by PG-RCA activated Cas12a. Target B molecules of different concentrations (indicated in the plot) were added as the primer of RL. Repeated measurements for the same reaction are plotted in the same color.

To evaluate the sequence design and determine whether CIRI can work simultaneously with PG-RCA, we carried out electrophoresis analysis for both DL and RL, with or without Cas12a-crRNA (Figure 2A). PG-RCA reactions were performed in buffer solution with a clean background, and target B was designed to trigger the amplification of RL. Reactions were performed at 37°C for only 30 min to avoid the generation of NBPs. Electrophoresis results show that amplicons of DL cannot activate Cas12a-crRNA, whereas amplicons of RL trigger the nonspecific trans-ssDNA cleavage that digests all amplicons produced by both circular templates.

We next evaluated the capability of Cas12a to disintegrate preassembled MNP clusters. Cas12a-based ternary complexes were prepared by mixing Cas12a with excess amounts of crRNAs and amplicons of RL, and were added into the preassembled MNP clusters to final Cas12a concentrations of 10, 30 and 100 nM. The suspension was incubated at 37°C and monitored by the optomagnetic sensor. Results are presented in terms of the change of the average phase lag (1–10 Hz) of the optomagnetic response after initiation of the reaction (see Supplementary Section S2 and Supplementary Figure S1). Figure 2B shows a time-resolved MNP cluster disassembly, demonstrating the dose-dependent catalytic ability of Cas12a. A Cas12a working concentration of 30 nM was chosen for the following tests. Next, we demonstrated that the catalytic ability of Cas12a could be activated by RL amplification. RL-based PG-RCA reactions were performed in the presence of RL (0.5 nM), preassembled MNP clusters, different concentrations of target B (as primers of RL) and Cas12a-crRNA complexes (30 nM). Figure 2C shows that activation of the Cas12a catalytic ability depends on the dose of target B that ranges from 100 fM to 100 aM. Optomagnetic signals remained constant at the beginning followed by an abrupt decrease due to the amplification of RL and the subsequent MNP cluster disassembly. Different concentrations of target B triggered signal reduction at different times, suggesting that CIRI can indicate amplification of RL during the PG-RCA reaction.

Assuming that NBPs are triggered by the presence of random nucleic acid sequences that can be contaminants, background nucleic acids and off-template products of polymerases, hybridization between those random sequences and circular templates can be regarded as a stochastic event influenced mainly by the temperature and their lengths (25). Therefore, we assume that nonspecific amplification reactions of both RL and DL in the same reaction suspension are stochastic events with very similar probabilities due to their identical length and GC content, thus the nonspecific activation of RL can be utilized to estimate the level of NBPs.

**Figure 3.**
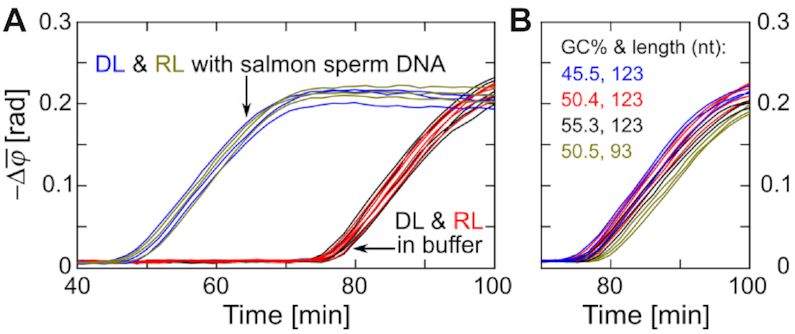
Optomagnetic performance of RL without Cas12a. (**A**) Real-time changes of $\bar{\varphi }$ (1–10 Hz) for the detection of background amplifications in clean buffer (*n* = 5) and in buffer containing nonspecific DNA (*n* = 3) using DL and RL. (**B**) Real-time changes of $\bar{\varphi }$ (1–10 Hz) for different RL designs. GC content and length of different RL designs are indicated in panel. Independent replicates are plotted in the same color.

To demonstrate that RL can indicate the background amplification of DL on time, template mixtures containing 0.5 nM each of DL and RL were amplified without target, Cas12a or crRNA (Figure 3A). The nonspecific PG-RCA reactions were performed both in clean target-free buffer and in buffer containing 0.1 μg/ml sheared salmon sperm DNA (1 nM, with an average size of 150 bp). To detect the background amplification of DL, MNPs in the suspension were modified with DP-DL-I and DP-DL-II oligos that can hybridize with amplicons of DL. For the detection of RL amplification, we used MNPs modified with DP-RL-I and DP-RL-II that can hybridize with amplicons of RL. As shown in Figure 3A, coincident results of DL and RL were observed both in clean target-free buffer (black and red curves) and in buffer containing nonspecific DNA (blue and yellow curves), suggesting that the activation of RL can indicate the background amplification of DL.

To investigate the influences of RL length and GC content on its ability to simulate the background amplification of DL, several RL designs were tested in clean buffer (Figure 3B). Real-time optomagnetic measurements show that GC content changes of ±5% have no obvious influence on RL performance (blue, red and black curves in Figure 3B), which confirms the low sequence-dependent amplification bias of RCA (14) and suggests a certain level of universality for the proposed RL design. Compared to the three 123-nt-long RLs, the 93-nt-long RL (yellow curves) exhibited ∼3 min delay of background amplification, which can be ascribed to the shorter binding sequence for nonspecific amplification triggers and/or the template length-dependent amplification bias of RCA (26).

Based on the probability assumption as well as experiment results, we herein summarize rules for RL design: First, RL should not hybridize with amplicons of DL at 37°C, with special attention to the flanking sequences of the nicking point (since both loop templates share the same nicking sequence). In this study, we chose Nb.BtsI with a nicking sequence 5′-NN^↓^CACTGC-3′. The 6-nt-long nicking sequence shared by amplicons of both DL and RL was located at their 5′-end to minimize the possibility of cross-hybridization since polymerase works at the 3′-end of the primer. Second, although it seems that there is tolerance to some variation in the GC content and length of the RL (Figure 3B), similar GC contents and lengths between RL and DL are preferred. Third, RL should contain secondary structures similar to DL, to ensure loop templates contain similar amounts of single-stranded areas that can bind freely to NBP triggers. In this work, secondary structures were investigated using NUPACK (www.nupack.org).

**Figure 4.**
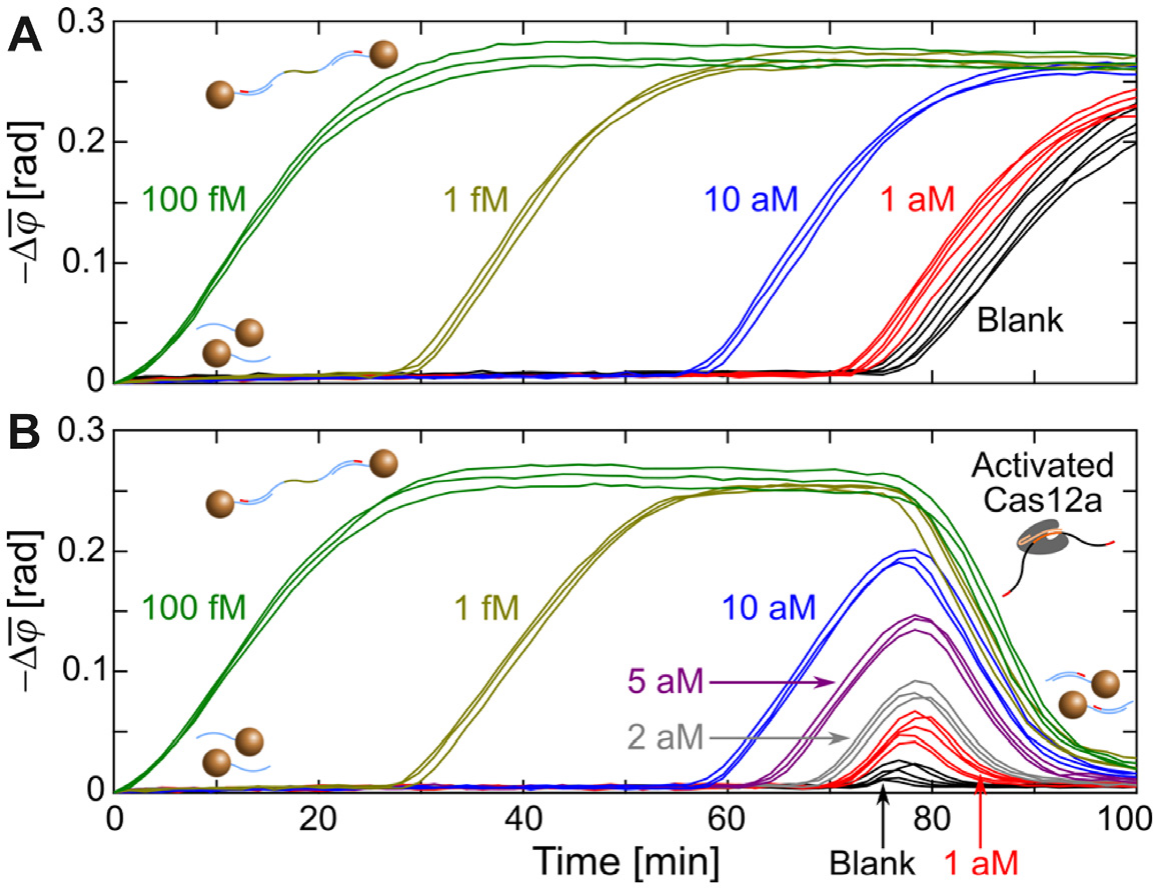
Real-time changes of $\bar{\varphi }$ (1–10 Hz) for the indicated target concentrations amplified by PG-RCA (**A**) without and (**B**) with CIRI. Independent replicates (*n* = 5 for 1 aM targets and blank controls, *n* = 3 for other target concentrations) are plotted in the same color. Black curves in Figures 3A and 4A are based on the same data.

Thereafter, we moved on to the RL amplicon-activated Cas12a system. PG-RCA reactions without and with CIRI were performed and monitored optomagnetically to demonstrate the capability of CIRI for indicating NBPs during nucleic acid amplification. Conventional PG-RCA reactions (without Cas12a) were performed to amplify target DNA of different concentrations. Real-time changes of $\bar{\varphi }$ (1–10 Hz) exhibited a time- and dose-dependence similar to the performance of other exponential nucleic acid amplification systems; the optomagnetic signal in each reaction remained constant at a baseline level in the early reaction stage but abruptly increased after a certain reaction time due to the amplification of DL and MNP clustering (Figure 4A). Independent replicates with the same target concentration provided very similar time-resolved curves. Blank controls provided similar spectra to those of the 1 aM samples (black and red curves in Figure 4A), suggesting that PG-RCA was initiated nonspecifically after *∼*70 min.

Real-time changes of $\bar{\varphi }$ (1–10 Hz) for different target concentrations amplified by CIRI-controlled PG-RCA are shown in Figure 4B. The early stages of the time-resolved spectra for reaction times below 70 min were nearly identical to those of PG-RCA without CIRI, suggesting that the presence of Cas12a-crRNA and RL did not have an observable influence on PG-RCA performance. As the reaction time increased, Cas12a was activated by NBPs produced by RL, leading to dramatic signal reductions. Remarkably, for weak positive samples and blank controls, onsets of specific amplicon-triggered MNP assembly and NBP-triggered cluster disassembly are close in time, resulting in peaks in the time-resolved spectra. The results suggested that CIRI could report onsets of nonspecific amplification in PG-RCA reactions. Based on the time-resolved spectra without and with CIRI, we infer that PG-RCA maintained its sensitivity and detection range when calculated based on a time threshold corresponding to the signal inflection points. In addition to the time threshold based analysis, the application of CIRI holds the potential to further increase PG-RCA performance by employing quantifications based on, e.g., the peak amplitude of the signal after onset of nonspecific amplification (Supplementary Section S3 and Supplementary Figure S2).

**Figure 5.**
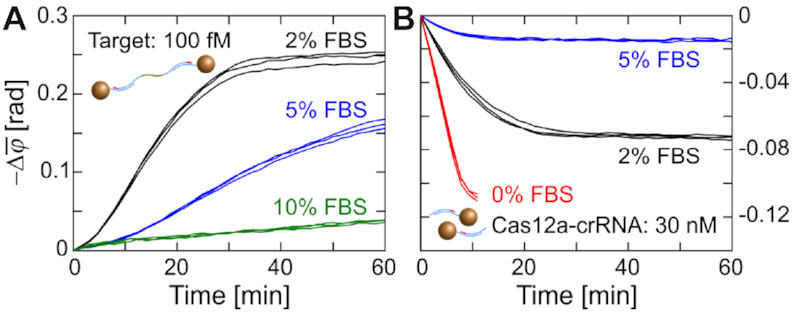
Performance of PG-RCA and CIRI in diluted FBS samples. Real-time changes of $\bar{\varphi }$ (1–10 Hz) are recorded for (**A**) PG-RCA of 100 fM target DNA and (**B**) cleavage of MNP clusters by preformed Cas12a ternary complexes. Final concentrations of FBS are indicated in panels. Independent replicates (*n* = 3) are plotted in the same color. Red curves in panel (**B**) are from Figure 2B for reference.

The stability of PG-RCA and CIRI against matrix effects was evaluated using diluted FBS (fetal bovine serum) samples. PG-RCA worked well in 2% FBS (final concentration) but was nearly inhibited in 10% FBS (Figure 5A). Stability of Cas12a and crRNA against FBS was evaluated by adding 30 nM of Cas12a ternary complexes into diluted FBS suspensions containing MNP clusters. The cleavage activity of Cas12a-based ternary complexes was lost within 20 min in 2% FBS (black curves in Figure 5B). This loss of activity was ascribed to the crRNA hydrolysis by serum nucleases. Although it was reported that chemical modification could make crRNAs stable at 37°C in 5% FBS for 30 min (27), the chemically modified crRNAs do still not meet our requirement since CIRI needs Cas12a-crRNA to stay activatable for at least 1.5 h. Therefore, additional reagents (RNase inhibitor) or processes (extraction or purification) have to be considered when testing serum samples using CIRI-controlled PG-RCA.

**Figure 6.**
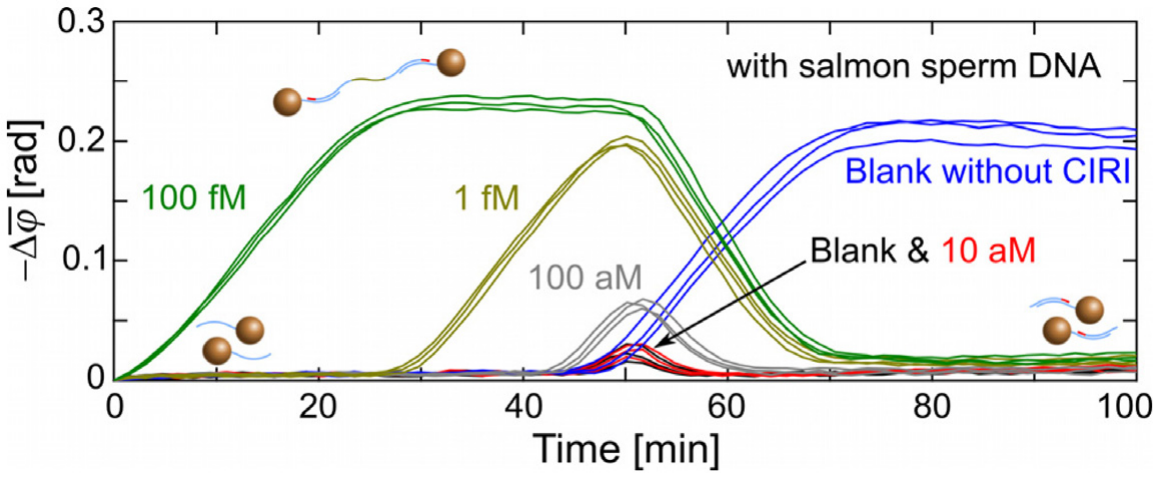
Real-time changes of $\bar{\varphi }$ (1–10 Hz) for the measurement of CIRI-controlled PG-RCA in samples containing 0.1 μg/ml sheared salmon sperm DNA. Independent replicates (*n*= 3) are plotted in the same color. Blue curves are from Figure 3A for reference.

The utility of CIRI was evaluated through PG-RCA reactions performed in the presence of 0.1 μg/ml sheared salmon sperm DNA (1 nM, with an average size of 150 bp) simulating the background of total DNA extraction samples (Figure 6). Due to the high DNA background, PG-RCA of blank controls was initiated earlier than in clean buffer (Figure 3A). Therefore, target concentrations <100 aM could hardly be distinguished from blank controls (cf. red and black curves in Figure 6). As expected, reactions performed with CIRI showed signal reductions after *∼*50 min, demonstrating the capability of CIRI to indicate the background DNA level as an internal negative control.

Although the capability of CIRI was demonstrated only in a PG-RCA strategy combined with an optomagnetic readout, it can be extended to other nucleic acid amplification strategies and readout methods. However, limitations have to be considered for applying CIRI. First, the reaction temperature has to be controlled near the optimal temperature of Cas12a. Second, similar to most of exponential amplification-based methodologies, CIRI requires or at least prefers a real-time (or time-resolved) readout not only for a wide dynamic detection range but also to avoid false-negative judgments (converted from false-positive judgments by CIRI). Third, the signal transducer (e.g. the MNP cluster) should be designed responsive to activated Cas12a. Fourth, the reference template has to be carefully designed to simulate the probability of detection template-based nonspecific amplification. Finally, it should be noted that there are two kinds of false-positive situations that cannot be detected by CIRI, (i) self-amplification of primers or primer dimers, which produces NBPs without the crRNA recognition sequence and (ii) amplification caused by carry-over contamination containing amplicons from previous reactions.

## CONCLUSION

In summary, we have developed an NBP-activated ssDNA-cutting approach that contains a reference template (RL), Cas12a, and its corresponding crRNA. The proposed approach, termed CIRI, can indicate the onset of nonspecific amplification when performed simultaneously with PG-RCA. Therefore, it can be utilized as an internal negative control to monitor the background level of amplification reactions in real-time, lowering the cost and simplifying the design of these methodologies.

## Supplementary Material

gkaa017_Supplemental_File
