# Supplementary material for: CRISPR-Cas12a based internal negative control for nonspecific products of exponential rolling circle amplification
Source: Nucleic Acids Res. 2020 Jan 20;48(5):e30. doi: 10.1093/nar/gkaa017 (PMC7049689; doi:10.1093/nar/gkaa017)
Supplement: gkaa017_Supplemental_File [file gkaa017_supplemental_file.docx]

**Supplementary Information**

CRISPR-Cas12a Based Internal Negative Control for Nonspecific Products of Exponential Rolling Circle Amplification

Bo Tian^1,^*, Gabriel Antonio S. Minero^1^, Jeppe Fock^2^, Martin Dufva^1^ and Mikkel Fougt Hansen^1,^*

^1^ Department of Health Technology, Technical University of Denmark, DTU Health Tech, Building 345C, DK-2800 Kongens Lyngby, Denmark

^2^ Blusense Diagnostics ApS, Fruebjergvej 3, DK-2100 Copenhagen, Denmark

* Corresponding authors. E-mail addresses: botia@dtu.dk; mfha@dtu.dk

**Table of Contents**

**Section S1.** Optomagnetic sensing theories.

**Section S2.** Optomagnetic biosensing principle.

**Figure S1.** Representative optomagnetic spectra of PG-RCA

**Section S3.** CIRI-controlled PG-RCA target quantification based on peak amplitude.

**Figure S2.** Dose-response of CIRI-controlled PG-RCA based on peak amplitude.

**Table S1.** DNA and RNA sequences used in this study.

**Section S1. Optomagnetic sensing theories**

The optomagnetic measurements were carried out in a previously described 4-chip optomagnetic setup (1). Briefly, in this setup, each chip containing a sample was sandwiched between two custom-built resistive heaters and the temperature controlled with an accuracy of about 1°C using Pt100 thermometers and a Stanford Research Systems PTC10 control unit. It took 1-2 minutes for the sample temperature to equilibrate after a change in temperature. Light from a light emitting diode ($=621 \mathrm{nm}$) was guided to the side of a chip and from the other side of a chip to a photodetector (Thorlabs PDA36A2) using rods of polymethyl-methacrylate (PMMA). In each setup, a magnetic field $B\left( t \right)=B_{0}\sin(2\pi ft)$ with $B_{0}=1 \mathrm{mT}$ applied along the light path was provided by two electromagnets supplied by a custom-built voltage-controlled current source. A data acquisition card (National Instruments, USB-6341) controlled the current to the electromagnets and recorded time traces of the photodetector voltages from the four setups from which the magnitude and phase of the signal harmonics with respect to the magnetic field excitation were extracted using a fast Fourier transformation algorithm. A spectrum consisting of 41 logarithmically equidistant frequencies between 1-2800 Hz was measured in *ca.* 50 s. The magnetic incubation was carried out in the setup using the same field generation coils, but with a higher value of the current.

The optomagnetic technique measures the time-dependent voltage from a photodetector illuminated through the nanoparticle sample by a light emitting diode in response to an applied oscillating magnetic field $B(t)=B_{0}\sin(2\pi ft)$. In our previous work (2–5), we have shown that the signal at low $B_{0}$ can be written as $V\left( t \right)=V_{\mathrm{ref}}+2V_{2}\sin^{2} (2\pi ft-\varphi)=V_{0}+V_{2}^{'}\sin(4\pi ft)+V_{2}^{''}\cos(4\pi ft)$ where $V_{\mathrm{ref}}$ is the signal in zero magnetic field, $V_{0}$ is the 0^th^ harmonic signal, $V_{2}$ is the amplitude of the 2^nd^ harmonic signal, $V_{2}^{'}=-V_{2}\sin(2\varphi)$ and $V_{2}^{''}=-V_{2}\cos(2\varphi)$. In these expressions $\varphi$ is the phase lag of the magnetic response, which increases with $f$ and reaches $\varphi=\pi/4$ at $f=f_{B}=k_{B}T/(\pi^{2}\eta D_{h}^{3})$, where $k_{B}T$ is the thermal energy, *η* is the viscosity and $D_{h}$ is the hydrodynamic diameter of the particle.

**Section S2. Optomagnetic biosensing principle**

Typical time-resolved optomagnetic spectra are shown in **Figure S1**. A positive sample containing 100 fM target DNA was PG-RCA (without CIRI) amplified as well as measured at 37°C for 60 min. **Figure S1a** and **b** show the typical spectra of the $V_{2}^{'}$ and the phase lag $\varphi$ of the magnetic response, respectively. During the amplification, MNPs were assembled by amplicons, resulting in the decrease of the negative peak located at *ca.* 80 Hz and the increase of the peak located at *ca.* 3 Hz (**Figure S1a**). The decreasing and increasing peaks of the $V_{2}^{'}$ spectra represent the reduction of the individual MNP concentration and the accumulation of MNP clusters, respectively. The decreasing $\varphi$-values at frequencies below about 30 Hz (**Figure S1b**) indicate MNP cluster assembly and that these show a signal of opposite sign of the individual MNPs in agreement with previous studies (3). To focus on the formation of MNP clusters, we calculated the average$\varphi$-value in the interval 1-10 Hz (grey zone in **Figure S1b**) denoted as $\overline{\varphi}$. The real-time signal changes, $-\Delta\overline{\varphi}$, from the $\overline{\varphi}$-value obtained after 1.5 min of reaction (measured after 20 magnetic incubation cycles to exclude the influences of temperature fluctuation and magnetic incubation-induced nonspecific MNP aggregation) were recorded to represent the degree of MNP assembly.


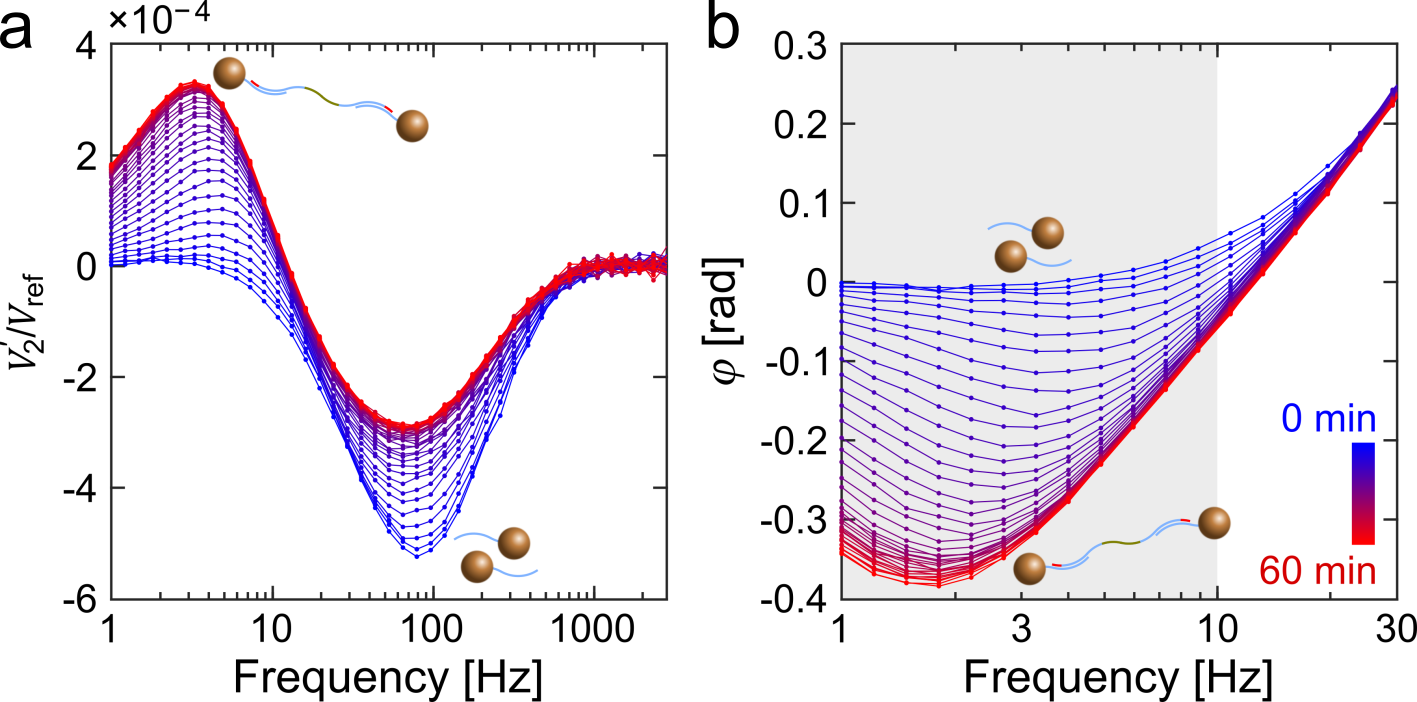


**Figure S1.** Representative optomagnetic spectra of PG-RCA for the detection of a positive control sample containing 100 fM target DNA. The blue curve indicates the initial spectrum while the red curve indicates the final spectrum. The grey zone indicates the frequency range used to calculate average $\varphi$ values.

**Section S3. CIRI-controlled PG-RCA target quantification based on peak amplitude**

The $-\Delta\overline{\varphi}$ peak amplitudes obtained after 70 min of CIRI-controlled PG-RCA were plotted against target concentrations of 1, 2, 5, and 10 aM. The cutoff value was calculated as the average peak amplitude plus three standard deviations of the blank controls. As shown in **Figure S2**, the $-\Delta\overline{\varphi}$ peak amplitude had a monotonic positive correlation with the target concentration between 1 aM and 10 aM, with coefficients of variation of 19.0%, 8.4%, 4.5% and 2.6%.


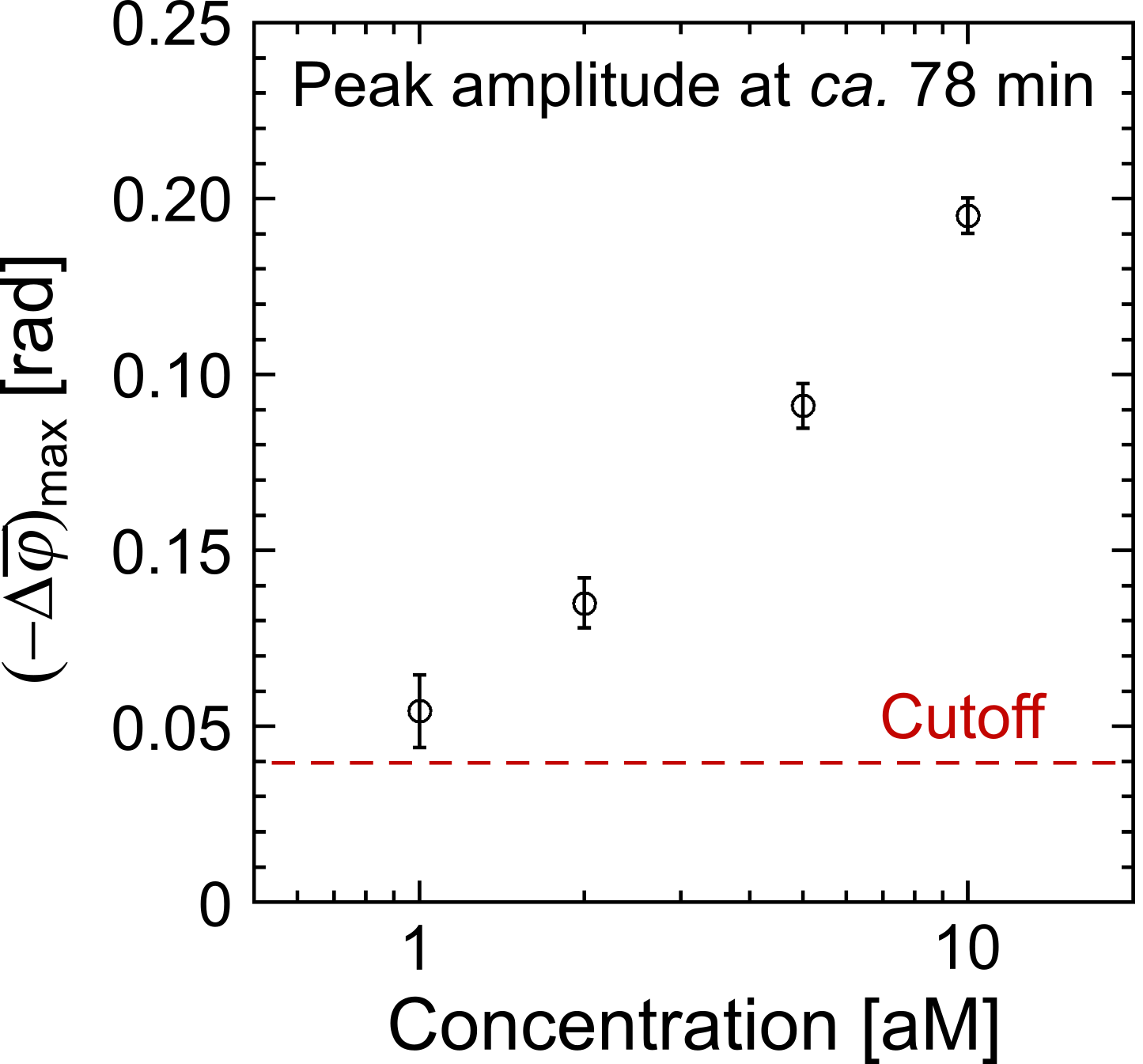


**Figure S2.** Dose-response of CIRI-controlled PG-RCA based on $-\Delta\overline{\varphi}$ peak amplitude. The red dashed line indicates the cutoff value. Spectra of these peaks can be seen in Figure 4b. Error bars indicate the standard deviations based on three independent replicates.

**Table S1.** DNA and RNA sequences. Recognition segments for Nb.BtsI are marked in red. Detection template binding area for target (DENV) is marked in yellow. For DL amplicon detection, DP-DL-I and DP-DL-II binding areas are marked in orange and purple, respectively. Reference template binding area for target B is marked in pink. The crRNA recognition segment and its corresponding segment in the reference template are marked in green. For RL amplicon detection, DP-RL-I binding area is underlined, and DP-RL-I binding area is **bold**.

| Name | Sequence (5'→3') |
| --- | --- |
| Target (DENV) | CTTTCAATATGCTGAAACGCGAGAGAAACCGCGT |
| Linear detection template | Phosphate-GGATAGCGTCATAGACGCGGTTTCTCTCGCGTTTCAGC  ATATTGAAAGCGTATAGTCACGCAGAGTCATCGTTCATCTAAGC  GAGTATGCGCAGTGATGCCATCGAGATCACGGCAACGGATG |
| Target B | ACTCTGTGCTAAGACCCTATCTGGCAGAGTGG |
| Linear reference template | Phosphate-ATGAGCAGTCAGCAGTG**CGACGTAACCACTCTGCCA**  **GATAGGGT**CTTAGCACAGAGTTCTCGACGACAACTTTAGATCG  TTACGCTAACTATGAGGGCTGTCTGTGGAATGCTACCTGAGGAG |
| crRNA | uaauuucuacucuuguagaugaucguuacgcuaacuauga |
| DP-DL-I | CAGAGTCATCGTTCATCTAAGCGAGTATGCTTTTTT-TEG-Biotin |
| DP-DL-II | Biotin-TEG-TTTTTTATGCCATCGAGATCACGGCAACGGATG-C3 |
| Linear reference template (high GC) | Phosphate-ATGAGCAGTCAGCAGTG**CGACGTAACCACTCTGCCA**  **GATAGGGT**CTCAGCACAGAGTTCTCGACGACCACTCCAGATCG  TGACGCTAACTATGAGGGCTGCCTGTGGAATGCTACCTGAGGAG |
| Linear reference template (low GC) | Phosphate-ATGAGCAGTCAGCAGTG**CGACGTAACCACTCTGCCA**  **GATAGGGT**CTTAGTACAGAGTTCTCGAAGACAACTTTAGATCG  TTATGCTAACTATGAGTTCTGTCTATGGAATGCTACCTGAGGAG |
| Linear reference template (short) | Phosphate-ATGAGCAGTCAGCAGTG**CGACGTAACCACTCTGCCA**  **GATAGGGT**CTTAGCACAGAGTTCTCGACGACAACTTTATGGAA  TGCTACCTGAGGAG |
| DP-RL-I | CCTGAGGAGATGAGCAGTCAGCAGTTTTTTT-TEG-Biotin |
| DP-RL-II | Biotin-TEG-TTTTTT**CGACGTAACCACTCTGCCAGATAGGGT**-C3 |

**REFERENCES**

1. Minero,G.A.S., Nogueira,C., Rizzi,G., Tian,B., Fock,J., Donolato,M., Strömberg,M. and Hansen,M.F. (2017) Sequence-specific validation of LAMP amplicons in real-time optomagnetic detection of Dengue serotype 2 synthetic DNA. *Analyst*, **142**, 3441–3450.

2. Mezger,A., Fock,J., Antunes,P., Østerberg,F.W., Boisen,A., Nilsson,M., Hansen,M.F., Ahlford,A. and Donolato,M. (2015) Scalable DNA-Based Magnetic Nanoparticle Agglutination Assay for Bacterial Detection in Patient Samples. *ACS Nano*, **9**, 7374–7382.

3. Fock,J., Parmvi,M., Strömberg,M., Svedlindh,P., Donolato,M. and Hansen,M.F. (2017) Comparison of optomagnetic and AC susceptibility readouts in a magnetic nanoparticle agglutination assay for detection of C-reactive protein. *Biosens. Bioelectron.*, **88**, 94–100.

4. Fock,J., Jonasson,C., Johansson,C. and Hansen,M.F. (2017) Characterization of fine particles using optomagnetic measurements. *Phys. Chem. Chem. Phys.*, **19**, 8802–8814.

5. Fock,J., Balceris,C., Costo,R., Zeng,L., Ludwig,F. and Hansen,M.F. (2018) Field-dependent dynamic responses from dilute magnetic nanoparticle dispersions. *Nanoscale*, **10**, 2052–2066.
